# PFKP binding AMOTL1 promotes tumor aerobic glycolysis and epithelial-mesenchymal transition by modulating Hippo pathway in head and neck cancer

**DOI:** 10.1515/jtim-2026-0006

**Published:** 2026-02-13

**Authors:** Lingwa Wang, Haiyang Li, Yifan Yang, Qian Shi, Ling Feng, Ru Wang, Jugao Fang

**Affiliations:** Department of Otorhinolaryngology Head and Neck Surgery, Beijing Tong Ren Hospital, Capital Medical University; Key Laboratory of Otorhinolaryngology Head and Neck Surgery, Ministry of Education, Beijing Institute of Otorhinolaryngology, Beijing, China

**Keywords:** phosphofructokinase platelet, aerobic glycolysis, head and neck cancer

## Abstract

**Background:**

Aerobic glycolysis drives cancer progression through phosphofructokinase (PFK)-mediated regulation. The contribution of platelet-type PFK (PFKP) to head and neck squamous cell carcinoma (HNSCC) pathogenesis remains undefined.

**Methods:**

Bioinformatic screening of 548 TCGA-HNSCC cases identified glycolysis-related prognostic genes *via* Cox regression, followed by experimental validation using quantitative polymerase chain reaction (qPCR) and Kaplan-Meier survival analysis in 51 clinical HNSCC tissue pairs in our center. *In vitro* functional assays (CCK8, colony formation, migration assays and metabolic analysis experiments), co-immunoprecipitation, ubiquitination analysis, and immunofluorescence were performed in HNSCC cell lines. PFKP-AMOTL1 interaction was validated by protein-binding studies. *In vivo* tumor growth was assessed using nude mice models. Clinical correlation analysis utilized HNSCC patient cohorts. Statistical significance was determined by Student's *t*-test and ANOVA.

**Results:**

PFKP emerged as the pivotal prognostic biomarker for HNSCC, demonstrating significant upregulation at mRNA/protein levels in tumors versus normal tissues (TCGA/clinical cohorts) and correlating with reduced overall and disease-specific survival. *In vitro*, PFKP overexpression enhanced aerobic glycolysis and epithelial-mesenchymal transition (EMT) in HNSCC cells. Mechanistically, PFKP directly bound AMOTL1, inhibiting its ubiquitin-mediated degradation. PFKP-driven glycolysis and EMT were AMOTL1-dependent. Furthermore, PFKP promoted YAP nuclear translocation *via* AMOTL1, suppressing Hippo pathway activity and amplifying glycolytic flux. *In vivo*, elevated PFKP accelerated tumor progression and glycolytic metabolism through AMOTL1/YAP/Hippo signaling.

**Conclusions:**

PFKP remodels tumor metabolism and drives EMT in HNSCC *via* the AMOTL1/YAP/Hippo axis, highlighting its mechanistic and prognostic significance, and suggesting its potential as a future therapeutic target.

## Background

Head and neck cancer has become the sixth most common malignancy worldwide, and its incidence has shown a significant increasing trend in recent years.^[1]^ Head and neck squamous cell carcinoma (HNSCC) is the most common histological type. According to global cancer statistics, HNSCC accounts for approximately 840,000 new cases annually. It is projected to rise to around 1 million cases by 2030, with an annual death toll of 450,000, ranking it twelfth.^[2,3]^ Pharyngeal cancer is a common and multiple malignant tumor in the head and neck region, accounting for over 60% of HNSCC cases. The characteristic of HNSCC is insidious onset and lack of specific symptoms in the early stage. More than 70% of patients are already in the local advanced stage at the time of diagnosis. Despite the existing comprehensive treatment system for tumors, the risk of local recurrence and distant metastasis remains high, ranging from 15% to 40%, with a 5-year overall survival (OS) rate of only about 25% to 35%.^[4, 5, 6]^ For a long time, tumor hypoxia and aerobic glycolysis have been considered as the main factors leading to treatment failure and disease progression in patients. Glycolysis not only rapidly provides energy for tumor cells but also supplies raw materials for the synthesis of various biomacromolecules, inhibits cell apoptosis, enhances the survival capacity of tumor cells under adverse conditions, and promotes the occurrence, development, migration, invasion, and drug resistance of tumors at different stages.^[7]^ Focusing on the aerobic glycolysis process of tumor cells and deeply exploring the metabolic changes and regulatory mechanisms of cancer cells can provide references for the basic research and clinical treatment of targeted cancer drugs.

Phosphofructokinase 1 (PFK1) is the major rate-limiting enzyme in the glycolytic pathway, catalyzing the conversion of fructose-6-phosphate to fructose-1, 6-bisphosphate. Phosphofructokinase platelet (PFKP) is the main isoform of PFK1 and mediates the catalytic reactions in the glycolytic pathway, serving as a critical regulator of cellular energy metabolism. Previous studies have demonstrated that PFKP, along with other metabolic enzymes, plays important roles in tumor progression, both in terms of metabolism and non-metabolism related functions, and is associated with poor prognosis. By modulating the expression or activity of these enzymes, cellular metabolic reprogramming can be influenced, providing growth advantages to tumor cells and promoting rapid proliferation, migration, invasion, as well as allowing tumor cells to evade immune cell killing, thus creating a favorable metabolic microenvironment for tumor progression.^[8,9]^ In glioblastoma cells, PFKP-dependent PI3K/AKT activation stabilizes PFKP and enhances the Warburg effect, while nuclear translocated pyruvate kinase M2 (PKM2) upregulates PFKP gene expression and glycolytic activity.^[10]^ Furthermore, in tumor cells, the key transcription factor Snail, involved in epithelial-mesenchymal transition, inhibits PFKP expression, promoting a shift from aerobic glycolysis to the pentose phosphate pathway. This metabolic reprogramming enables tumor cells to evade normal apoptotic processes and facilitates proliferation and migration.^[11]^ Under glucose deprivation conditions, PFKP interacts with AMP-activated protein kinase (AMPK), enhancing mitochondrial recruitment of AMPK, which in turn phosphorylates and activates ACC2, thereby promoting fatty acid oxidation for energy production, alleviating glucose deprivation-induced metabolic stress, maintaining energy homeostasis in lung cancer cells, and promoting their survival.^[12]^ However, the functional mechanisms of PFKP in the development and progression of HNSCC remain largely unknown.

In this study, we identified PFKP as the central factor promoting the tumorigenesis and affecting HNSCC patients’ prognosis through comprehensive analysis of bioinformatic data from the TCGA database and our own samples. Then, we investigated the effects of PFKP expression changes on HNSCC cell proliferation, migration, epithelial-mesenchymal transition, and aerobic glycolysis through cellular functional experiments. Subsequently, we explored the downstream molecules of PFKP and their interaction mechanisms through molecular biology experiments. Furthermore, we validated the downstream pathway of PFKP in nude mice.

Importantly, the role of metabolic reprogramming extends beyond fueling tumor growth. The glycolytic phenotype, characterized by enzymes like PFKP, leads to lactate production and acidification of the tumor microenvironment (TME), which is known to foster immunosuppression and confer resistance to conventional therapies and immunotherapy.^[13]^ Furthermore, key oncogenic pathways driving HNSCC, such as the MEK/ERK cascade, can be activated by diverse stimuli including metabolic alterations and exogenous factors like nicotine, highlighting the interconnectedness of metabolism and established progression mechanisms.^[14]^ Therefore, elucidating the function of PFKP in HNSCC is critical not only for understanding metabolic rewiring but also for deciphering its potential role in modulating the TME and therapeutic response.

## Methods

### Cell lines and cell transfection

Human pharyngeal squamous cell carcinoma (Fadu), laryngeal squamous cell carcinoma (TU138), and tongue squamous cell carcinoma (Cal27) cell lines were obtained from the Beijing Institute of Otorhinolaryngology (Beijing, China) and authenticated by STR profiling within the past three years. Fadu and Cal27 cells were cultured in Dulbecco’s Modified Eagle Medium (DMEM, Gibco, catalog number: C1995500BT) supplemented with 10% fetal bovine serum (FBS, Gibco, catalog number: 10099141C) at 37 °C in a 5% CO_2_ humidified incubator. TU138 cells were maintained in Roswell Park Memorial Institute (RPMI) 1640 medium (Gibco, catalog number: 22400089) with 10% fetal bovine serum.

For functional studies and rescue experiments, plasmids for overexpression of PFKP (OE-PFKP), knockdown of PFKP (shPFKP), overexpression of AMOTL1 (OE-AMOTL1), knockdown of AMOTL1 (siAMOTL1), and knockdown of YAP (siYAP) were constructed, along with their corresponding negative control plasmids (shCtrl, OE-Ctrl). Transfections were performed using Polyethylenimine linear (PEI, Sigma, catalog number: 408727) according to the manufacturer’s protocol. Successful knockdown and overexpression were confirmed by qPCR) after 72 h of transfection.

OE-PFKP-F: 5’-GGTGAATTCCTCGAGACTAGTTCTAGAGCCACCATGGACGCGGACGACT CCCGG-3’, OE-PFKP-R: 5’-GGAGGGAGAGGGGCGGGATCCGCGGCCGCCTACTTGTCATCGTCGTCCTTGTAATCGACACTCCAGGGCTGCACAT-3’, shPFKP-F: 5’-CCGGCTGAACACCTACAAGCGACTTC TCGAGAAGTCGCTTGTAGGTGTTCAGTTTTTG-3’, shPFKP-R: 5’-AATTCAAAAACTGAACACCTACAAGCGACTTCTCGAGAAGTCGCTTGTAGGTGTTCAG-3’, siAMOTL1: 5’-CACACAGACTGACAAGAGT-3’, siYAP: 5’-CCACCAAGCTAGATAAAGA-3’

### CCK8, colony formation and migration assays

Cell proliferation, colony formation, and migration assays were performed to evaluate cell behavior. For proliferation, cells were seeded at 10,000 cells per well in 96-well plates with 100 μL culture medium. At 1, 2, 3, and 4 days, 10 μL of CCK-8 solution (MCE, catalog number: HY-K0301) was added, and cells were incubated for 1 h at 37 °C. Absorbance was measured at 450 nm. For colony formation, 500 cells were plated in 6-well plates and cultured for 10–14 days with medium changes every 3 days. Colonies were fixed with 4% paraformaldehyde for 10 min, stained with 0.1% crystal violet for 15 min, and colonies containing more than 50 cells were counted microscopically. For migration, 20,000 cells in 200 μL serum-free medium were seeded into the upper chamber of transwell plates (8.0 μm pore size, Corning, catalog number: 3422), and 600 μL medium containing 10% fetal bovine serum was added to the lower chamber as a chemoattractant. After 24 h of incubation at 37 °C, cells on the lower membrane were fixed, stained, and counted under a microscope. Each assay was performed in triplicate to ensure reproducibility.

### Metabolic analysis experiments

Lactate and ATP levels were quantified using Solarbio assay kits (catalog numbers BC5345 and BC0300, respectively). After centrifugation, 10,000 cells were collected and incubated with assay reagents. Absorbance was measured at 450 nm for lactate and 340 nm for ATP using a microplate reader, and concentrations were calculated using standard curves.

Seahorse metabolic assays were conducted using a Seahorse Biosciences XF96 analyzer (North Billerica, MA, USA). The Seahorse XF Glycolysis Stress Test Kit (Agilent, catalog number: 103020-100) was used according to the manufacturer’s instructions. On the day of plating, 180 μL of hydration solution was added to the lower wells of the XF96 Extracellular Flux Assay Kits and incubated overnight at 37 °C in a non-CO_2_ incubator. The required drugs were prepared and mixed with Seahorse XF Base Medium, adjusting the pH to 7.4. The following day, cells were washed twice with pre-warmed Seahorse XF Base Medium, and 175 μL of this medium was added to each well. Plates were incubated for 1 h at 37 °C without CO_2_. Drugs were diluted to the desired concentrations and added to the upper wells (25 μL per well). After 30 min, the equilibrated plates were placed in the analyzer for measurement. Extracellular acidification rate (ECAR) was used to assess glycolytic capacity, while oxygen consumption rate (OCR) reflected mitochondrial function.

### Western blot

Western blotting was used to assess protein expression under various transfection conditions. Cell lysates were prepared in 2× SDS sample buffer (100 mmol/L Tris-HCl, pH 6.8; 10 mmol/L EDTA; 4% SDS; 10% glycerol). Equal protein amounts (20 μg) were separated by SDS-PAGE and transferred to a PVDF membrane. The membrane was blocked with 5% milk, incubated with primary antibodies, followed by secondary antibodies, and protein bands were detected using an ECL detection system (Amersham). Actin and GAPDH served as loading controls. Band intensities were quantified using an ImageQuant densitometer (Molecular Dynamics). All experiments were performed in triplicate. The details of antibodies used in the experiment are shown in Supplementary Table S1.

### Immunoprecipitation (IP) and mass spectrometry

For the IP assays, cells were lysed on ice for 30 min using NP-40 lysis buffer (Beyotime, P0013F) supplemented with protease inhibitor cocktail (Roche, 4693159001). Following centrifugation, total protein concentration in the supernatant was quantified *via* BCA assay. For each reaction, 500–800 μg of protein lysate was incubated with 2–4 μg of PFKP antibody or equivalent normal IgG as an isotype control overnight at 4 °C with gentle rotation. Subsequently, immune complexes were captured by incu bation with 30 μL of pre-washed Protein A/ G Magnetic Beads (MedChemExpress, HY-K0202) for 4 h at 4 °C. The beads were then extensively washed five times with ice-cold lysis buffer, and the bound proteins were finally eluted by boiling in 1× SDS-PAGE loading buffer at 95 °C for 10 min prior to downstream analysis.

For mass spectrometry analysis, the eluted proteins were separated by SDS-PAGE on a 10% gel. The entire lane was excised and subjected to in-gel tryptic digestion. The resulting peptides were analyzed by liquid chromatography-tandem mass spectrometry (LC-MS/MS) on a Q Exactive HF mass spectrometer (Thermo Fisher Scientific) coupled to an EASY-nLC 1200 system. The raw data were searched against the Swiss-Prot human database using the MaxQuant software (version 2.0.3.0). Proteins identified in the PFKP-IP group but not in the IgG control group, with at least two unique peptides and a false discovery rate (FDR) < 1%, were considered high-confidence interacting partners.

### Exogenous co-immunoprecipitation (Co-IP) validation

To validate protein-protein interactions, exogenous Co-IP was performed after transient transfection of cells with plasmids encoding tagged proteins. Forty-eight hours post-transfection, cell lysates were prepared in lysis buffer and incubated with PFKP antibody or control IgG. Immunocomplexes were captured using protein A/G beads, extensively washed, and analyzed by Western blotting to detect co-precipitated proteins.

### Ubiquitination degradation assay

Cells were treated with cycloheximide (CHX, 50 μg/mL, MedChemExpress, catalog number: HY-12320) to inhibit protein synthesis. At the indicated time points, cells were harvested, and lysates were prepared in lysis buffer containing protease inhibitors (Roche, catalog number: 4693159001). Immunoprecipitation was performed using antibodies against the target protein, and ubiquitinated proteins were detected with anti-ubiquitin antibodies. Target protein degradation was monitored by Western blotting at various time points.

### RNA sequencing and pathway enrichment analysis

Total RNA was extracted from PFKP knockdown and control cells using TRIzol reagent (Invitrogen, catalog number: 15596026). RNA quality was assessed prior to transcriptome sequencing. Differential gene expression (DEG) analysis was performed using DESeq2, with genes exhibiting an adjusted *P*-value < 0.05 considered significant. Pathway enrichment analysis was conducted using the ClusterProfiler package in R.

### Immunofluorescence staining and confocal microscopy analysis

Cells were transfected for 24 h, fixed with 4% paraformaldehyde (PFA, Solarbio, catalog number: P 1110) for 15 min, and permeabilized with 0.1% Triton X-100 in 5% BSA-PBS for 10 min. After blocking with 5% BSA in PBS for 1 h, primary antibodies, diluted in 1% BSA-PBS, were applied for 2 h at room temperature. After washing, fluorophore-conjugated secondary antibodies were applied and incubated for 1 h in the dark. Nuclei were stained with DAPI for 5 min. Immunofluorescent images were acquired using a Leica TCS SP5 confocal microscope, with appropriate laser settings and filters. Images were processed using Leica Application Suite software, and fluorescence intensity and protein localization were analyzed to assess expression and cellular distribution. Quantification of protein nuclear translocation was performed using ImageJ software. Briefly, the nucleus was selected as the region of interest (ROI) based on the DAPI signal. A peripheral annular area surrounding the nucleus was selected as the cytoplasmic ROI. The fluorescence intensity of the target protein was measured in the nuclear and cytoplasmic ROIs for each individual cell. The nuclear-to-cytoplasmic (N/C) fluorescence intensity ratio was then calculated. Statistical analysis was performed using a *t*-test to compare the differences between groups.

### Clinical specimens

A total of 51 pairs of tumor and adjacent normal tissue samples were collected from patients diagnosed with HNSCC between May 2020 and May 2023. The study protocol was approved by the institutional review boards of all participating centers, with central approval granted by the ethics committee of Beijing Tongren Hospital (Approval No. TREC2022-KY018. R1), and written informed consent was obtained from all participants. Inclusion criteria were: (1) histologically confirmed primary HNSCC; (2) age 18 to 75 years; (3) adequate hematologic, hepatic, and renal function. Key exclusion criteria included: (1) evidence of distant metastasis (M1); (2) history of another primary malignancy within the past 5 years;(3) prior radiotherapy to the head and neck region; or (4) any condition precluding protocol-specified treatment. The cohort included patients with a median age of 62 years (range 41–73]). The detailed clinicopathological characteristics of all patients, including age, gender, smoking history, TN stage, and clinical stage, are summarized in Supplementary Table S2.

RNA was extracted from these samples for qPCR analysis to assess target gene expression. Prognostic differences between high and low expression groups were evaluated using Kaplan-Meier survival analysis. The use of human HNSCC tissues and informed consent was approved by the hospital’s ethics board. All experiments were performed in duplicates, with triplicates for each sample. Data are presented as mean ± standard deviation (SD). Target protein expression was assessed by immunohistochemistry, and co-expression was analyzed through tissue co-localization.

PFKP-F: 5’-CGGAAGTTCCTGGAGCACCTCTC-3’

PFKP-R: 5’-AAGTACACCTTGGCCCCCACGTA-3’

GAPDH-F: 5’-ACCACAGTCCATGCCATCAC-3’

GAPDH-R: 5’-TCCACCACCCTGTTGCTGTA-3’

AMOTL1-F: 5’-CGGGGAACTTGTGAGCCTG-3’

AMOTL1-R: 5’-CTGGGGAAAAGTAGGTGGAGT-3’

### Nude mouse tumorigenesis experiment

Nude mice were subcutaneously implanted with tumor cells and monitored for growth. Tumor volume was measured every three days using the formula: volume = (length × width^2^) /2, and tumor mass was recorded at euthanasia. Ki-67 expression in tumor tissues was assessed by fixing in 4% paraformaldehyde, embedding in paraffin, sectioning, and performing immunohistochemistry (IHC) with a Ki-67 antibody, followed by secondary antibody incubation and DAB development. Western blotting was used to evaluate downstream target protein expression. Tumor volume, mass, Ki-67 expression, and protein levels were compared across groups to assess treatment effects on tumor growth and molecular signaling.

## Results

### PFKP is identified as the pivotal prognostic factor for HNSCC

**Figure 1:**
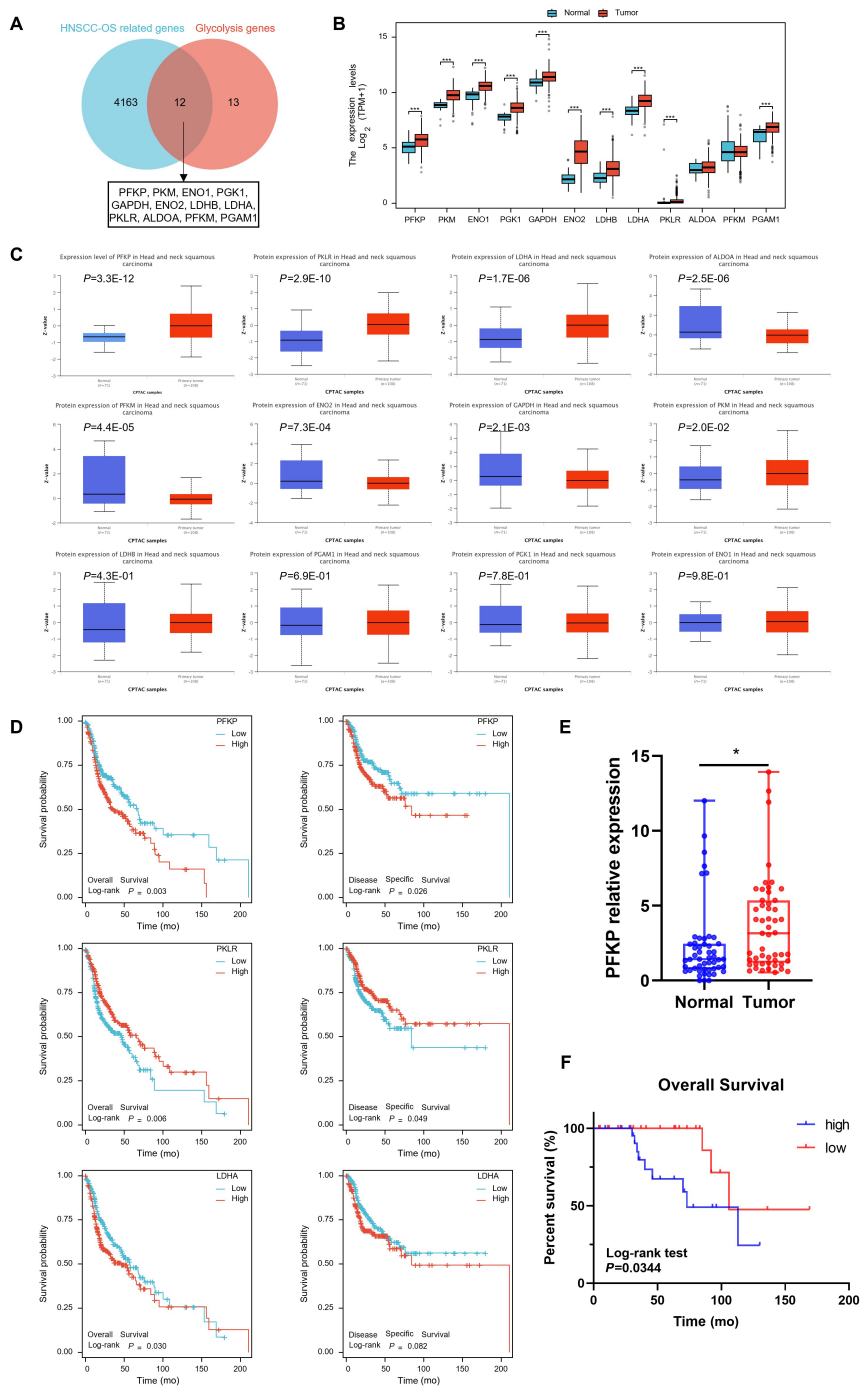
Identification of the hub prognostic factor for HNSCC. A. The Venn diagram shows the intersection of the significant genes related to overall survival from the TCGA database and glycolysis genes from the KEGG database. B. Analysis of mRNA level differences of 12 hub genes between cancer and adjacent normal tissues using the TCGA database. C. Analysis of protein level difference of 12 hub genes between cancer and adjacent normal tissues in CPTAC database. D. Kaplan-Meier plot was generated to assess the overall survival and disease specific survival of HNSCC patients in the TCGA dataset based on PFKP, PKLR and LDHA expression. E. The qPCR analysis of mRNA level differences of PFKP between cancer and normal tissues was performed in our center. F. Kaplan-Meier survival analysis indicated that the prognosis of patients with high PFKP expression group was worse. ^*^P < 0.05, ^***^P < 0.001.

A total of 548 patients from the TCGA public database with RNA-seq data were included to screen important genes related to prognosis. We found 4163 significant genes related to OS by cox regression. Additionally, we retrieved a compilation of 25 glycolysis genes from the *KEGG* (Kyoto Encyclopedia of Genes and Genomes) database. The intersection of these two gene sets comprised PFKP, PKM, ENO1, PGK1, GAPDH, ENO_2_, LDHB, LDHA, PKLR, ALDOA, PFKM, and PGAM (Figure 1A). Notably, analysis of the TCGA database revealed upregulation of PFKP, PKM, ENO1, PGK1, GAPDH, ENO_2_, LDHB, LDHA, PKLR, and PGAM mRNA levels in HNSCC (Figure 1B). Compared with normal tissues, only the protein level of PFKP, PKLR and LDHA are significantly increased in HNSCC (Figure 1C). Therefore, we continue to analyze the influence of the expression level of these three factors on the prognosis of HNSCC patients. Further survival analysis revealed a significantly lower OS rate and disease specific survival (DSS) rate among patients with high PFKP expression (Figure 1D), which is consistent with our previous findings.

To validate these findings, we collected 51 pairs of tumor and adjacent tissue samples from HNSCC patients in our center. QPCR analysis confirmed significantly higher expression of PFKP in tumor tissue es compared to normal tissues (Figure 1E). Kaplan-Meier survival analysis showed that the prognosis of patients with high PFKP expression group was worse (Figure 1F). Collectively, these data demonstrate that PFKP serves as the pivotal prognostic factor for HNSCC.

### PFKP promotes aerobic glycolysis and EMT in HNSCC cells

**Figure 2:**
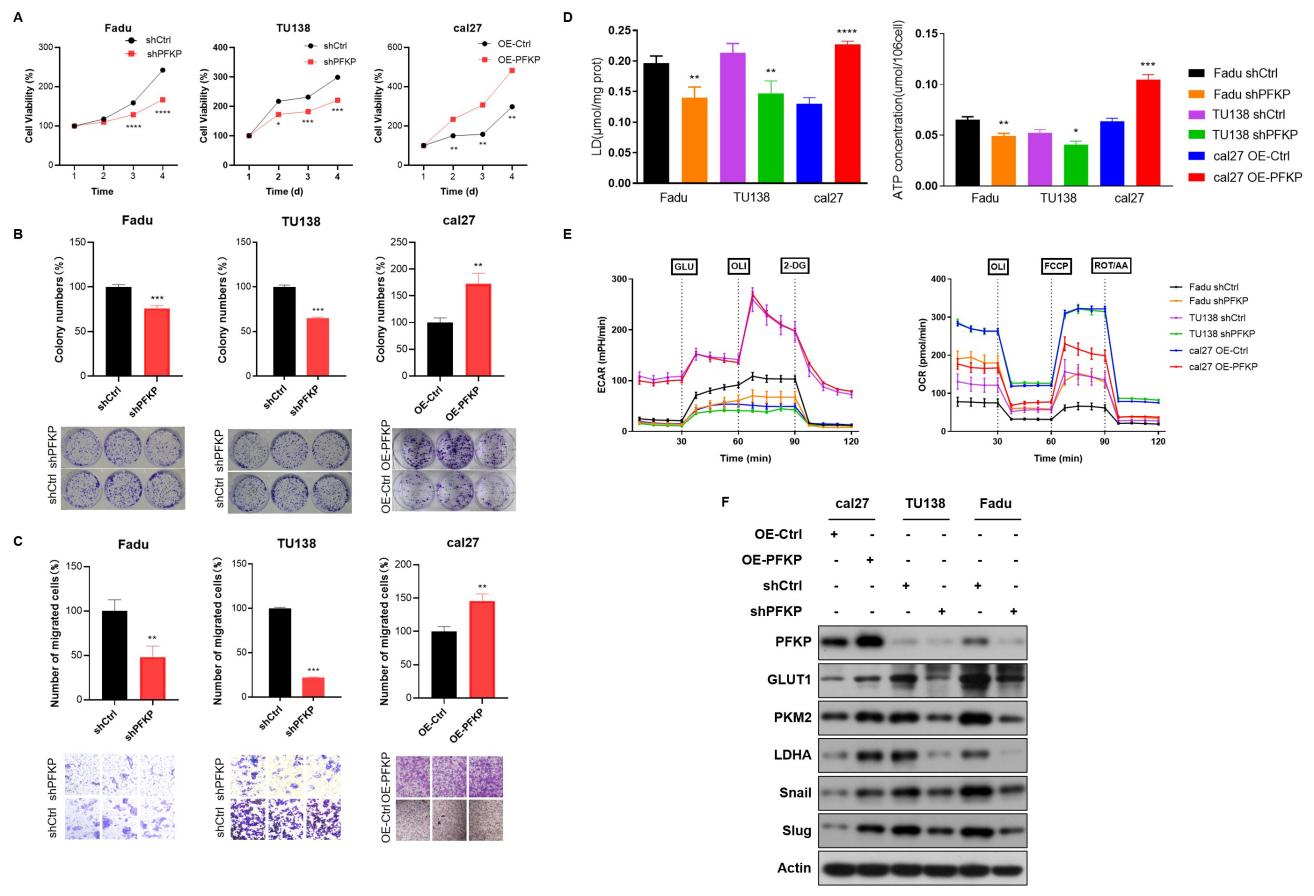
PFKP promotes proliferation, migration, aerobic glycolysis and epithelial-mesenchymal transition (EMT) in HNSCC cells. A. Cell growth rates were assessed in PFKP-knockdown Fadu and TU138 cells, as well as PFKP-overexpressing cal27 cells using CCK-8 assays. B. Colony-formation assays were employed to investigate the impact of PFKP knockdown and overexpression on colony-forming ability. C. Migration rates of PFKP-knockdown and overexpressing cells were determined using transwell assays. D. Lactate and ATP levels were measured in PFKP-knockdown and overexpressing cells. E. Seahorse metabolic analysis was conducted to assess extracellular acidification rate (ECAR) and oxygen consumption rate (OCR). F. Western blot analysis was performed to examine the expression of glycolysis-related proteins GLUT1, PKM2, LDHA and EMT-related molecules Snail and Slug in PFKP-knockdown and overexpressing cells. Data are presented as mean ± SD. ^*^P < 0.05, ^**^P < 0.01, ^***^P < 0.001, ^****^P < 0.0001.

To assess the impact of PFKP expression on cellular processes in HNSCC cells, we manipulated PFKP levels and conducted a series of experiments. The CCK8 assay revealed that PFKP knockdown reduced Fadu and TU138 cells proliferation, while PFKP overexpression enhanced it in cal27 cells (Figure 2A). Colony formation assays further confirmed reduced colony formation ability in PFKP knockdown group and increased ability in PFKP overexpression group (Figure 2B). Additionally, we investigated the migration ability of HNSCC cells and found that knockdown of PFKP decreased migration quantity, whereas overexpression of PFKP increased migration (Figure 2C). Furthermore, we examined the effects of PFKP expression on lactate production and ATP levels. Knockdown of PFKP significantly decreased lactate and ATP levels, while overexpression of PFKP significantly increased these levels (Figure 2D). Seahorse metabolic analysis further revealed that PFKP knockdown resulted in a reduction in ECAR and an increase in OCR, while overexpression of PFKP promoted ECAR and inhibited OCR (Figure 2E, Supplementary Figure S1). Western Blot analysis validated our findings by demonstrating decreased expression of glycolysis-related proteins GLUT1, PKM2, LDHA, as well as epithelial-mesenchymal transition (EMT)-related molecules Snail and Slug upon PFKP knockdown, and increased expression upon PFKP overexpression (Figure 2F). Overall, our results highlight the significant role of PFKP in promoting proliferation, migration, EMT, and aerobic glycolysis in HNSCC cells.

### Interaction between PFKP and AMOTL1 suppress the ubiquitination degradation of AMOTL1

**Figure 3:**
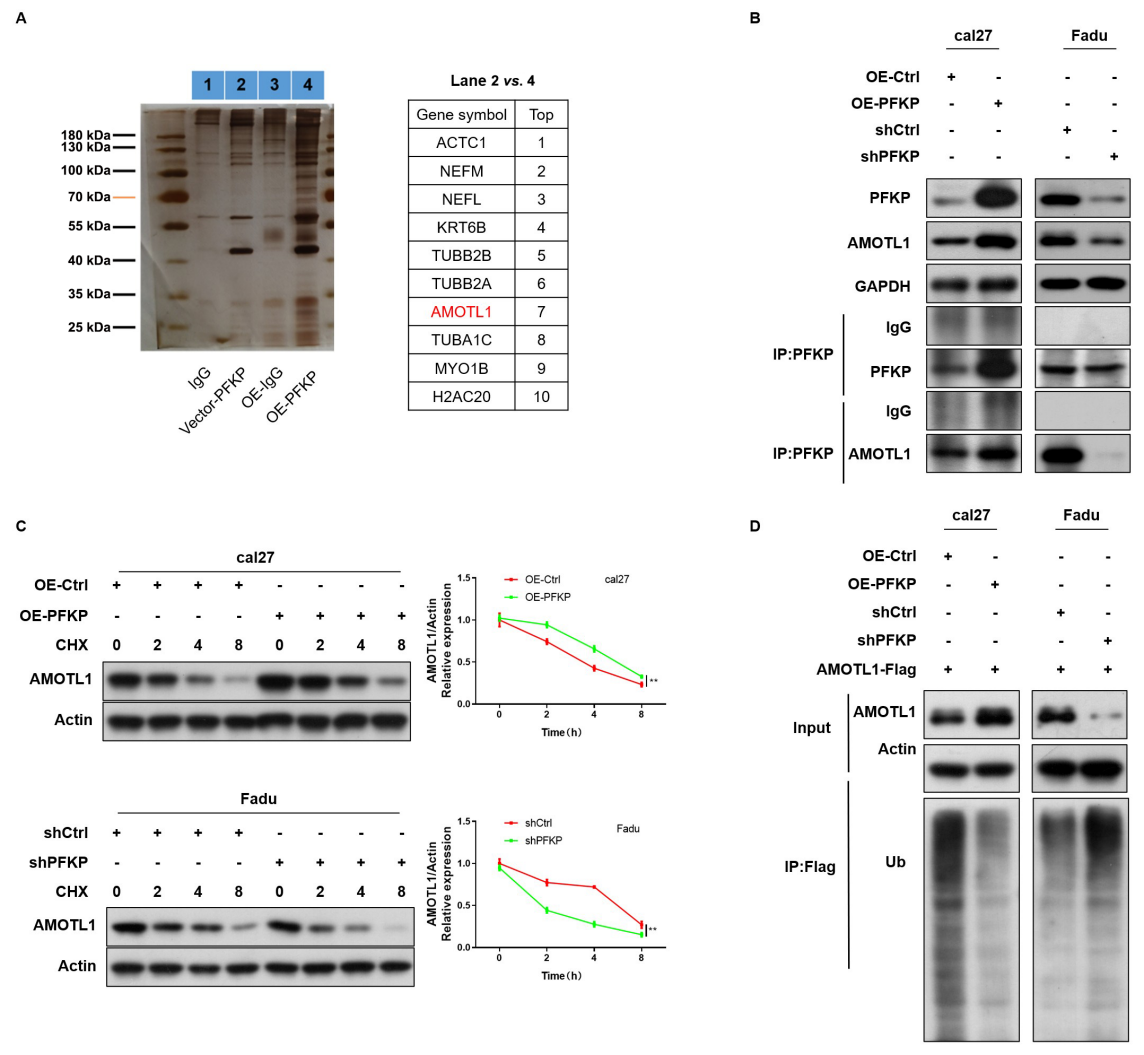
Interaction between PFKP and AMOTL1 inhibits the ubiquitination-mediated degradation of AMOTL1. A. Mass spectrometry analysis identified the target protein of PFKP. B. Exogenous Co-IP validated the direct interaction between PFKP and the AMOTL1. C. protein half-life experiments determined the degradation rate of protein AMOTL1 in PFKP-knockdown and overexpressing cells. D. The ubiquitination level of AMOTL1 was assessed in PFKP-knockdown and overexpressing cells. Data are presented as mean ± SD. ^**^P < 0.01.

After performing the IP experiment, the precipitated proteins were visualized on SDS-PAGE using silver staining. Mass spectrometry identified the top ten proteins (ACTC1, NEFM, NEFL, KRT6B, TUBB2B, TUBB2A, AMOTL1, TUBA1C, MYO1B, H2AC20) interacting with PFKP (Figure 3A). Exogenous Co-IP validation confirmed the direct interaction between PFKP and AMOTL1 protein. Overexpressing PFKP increased co-precipitated AMOTL1 protein, whereas knockdown of PFKP decreased it (Figure 3B). Next, we examined whether PFKP's influence on AMOTL1’s degradation through protein half-life experiments. The protein level of AMOTL1 gradually decreased with increasing exposure time to CHX (Figure 3C). Overexpression of PFKP slowed down the degradation rate of AMOTL1, while knockdown of PFKP accelerated it (Figure 3C). AMOTL1 belongs to the angiomotin family of binding proteins, primarily modified by ubiquitination. Overexpression of PFKP significantly reduced AMOTL1’s ubiquitination level compared to the control group, while knockdown of PFKP increased it (Figure 3D). These findings indicated that higher PFKP expression weakens the ubiquitination of AMOTL1. Our results demonstrate the direct interaction between PFKP and AMOTL1. PFKP inhibits AMOTL1’s ubiquitination, thereby suppressing its degradation.

### PFKP promotes EMT and aerobic glycolysis dependent on AMOTL1

**Figure 4:**
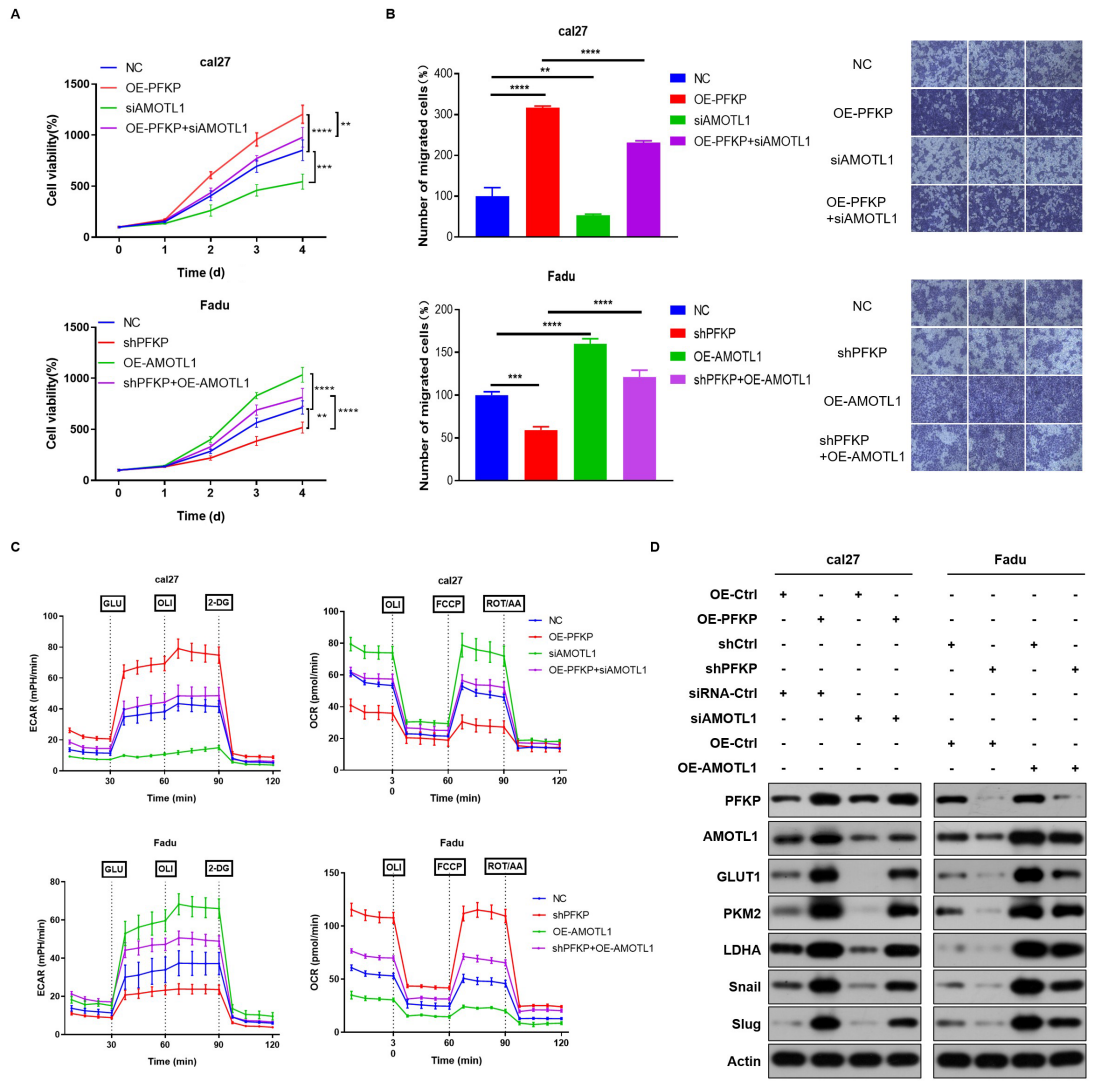
PFKP enhances tumor cell proliferation, EMT and aerobic glycolysis through AMOTL1. A. Cell proliferation was assessed in Fadu and cal27 cells by performing the CCK-8 assay simultaneously overexpressing PFKP and knockdown AMOTL1, or knockdown PFKP and overexpressing AMOTL1. B. Transwell assays were performed with simultaneous overexpression of PFKP and knockdown of AMOTL1, or simultaneous knockdown of PFKP and overexpression of AMOTL1 to evaluate cell migration. C. Seahorse metabolic analysis was conducted to investigate the effect of PFKP-dependent AMOTL1 on aerobic glycolysis. D. Western Blot was employed to assess the expression of glycolytic metabolism-related proteins and EMT-related molecules. Data are presented as mean ± SD. ^**^P < 0.01, ^***^P < 0.001, ^****^P < 0.0001.

We conducted recovery experiments to investigate the impact of PFKP on tumor cell proliferation, EMT and aerobic glycolysis mediated by AMOTL1. Simultaneous overexpression of PFKP and knockdown of AMOTL1 significantly reduced the proliferative and migratory ability of cal27 cells. Conversely, simultaneous knockdown of PFKP and overexpression of AMOTL1 increased the proliferative and migratory activity of Fadu cells (Figure 4A, B). Seahorse experimental results demonstrated that knocking down AMOTL1 partially reversed the role of PFKP in promoting glycolysis and inhibiting mitochondria, while overexpressing AMOTL1 partially reversed the effects of PFKP knockdown in inhibiting glycolysis and promoting mitochondria (Figure 4C, Figure S2). Moreover, Western Blot results revealed that knockdown of AMOTL1 rescued the expression of glycolytic metabolism-related proteins and EMT-related molecules affected by PFKP overexpression. Conversely, overexpression of AMOTL1 increased the expression of these proteins due to PFKP knockdown (Figure 4D). These findings indicate that PFKP promotes tumor cell proliferation, EMT and aerobic glycolysis in an AMOTL1-dependent manner.

### PFKP targets AMOTL1 to promote YAP nuclear translocation in HNSCC cells

**Figure 5:**
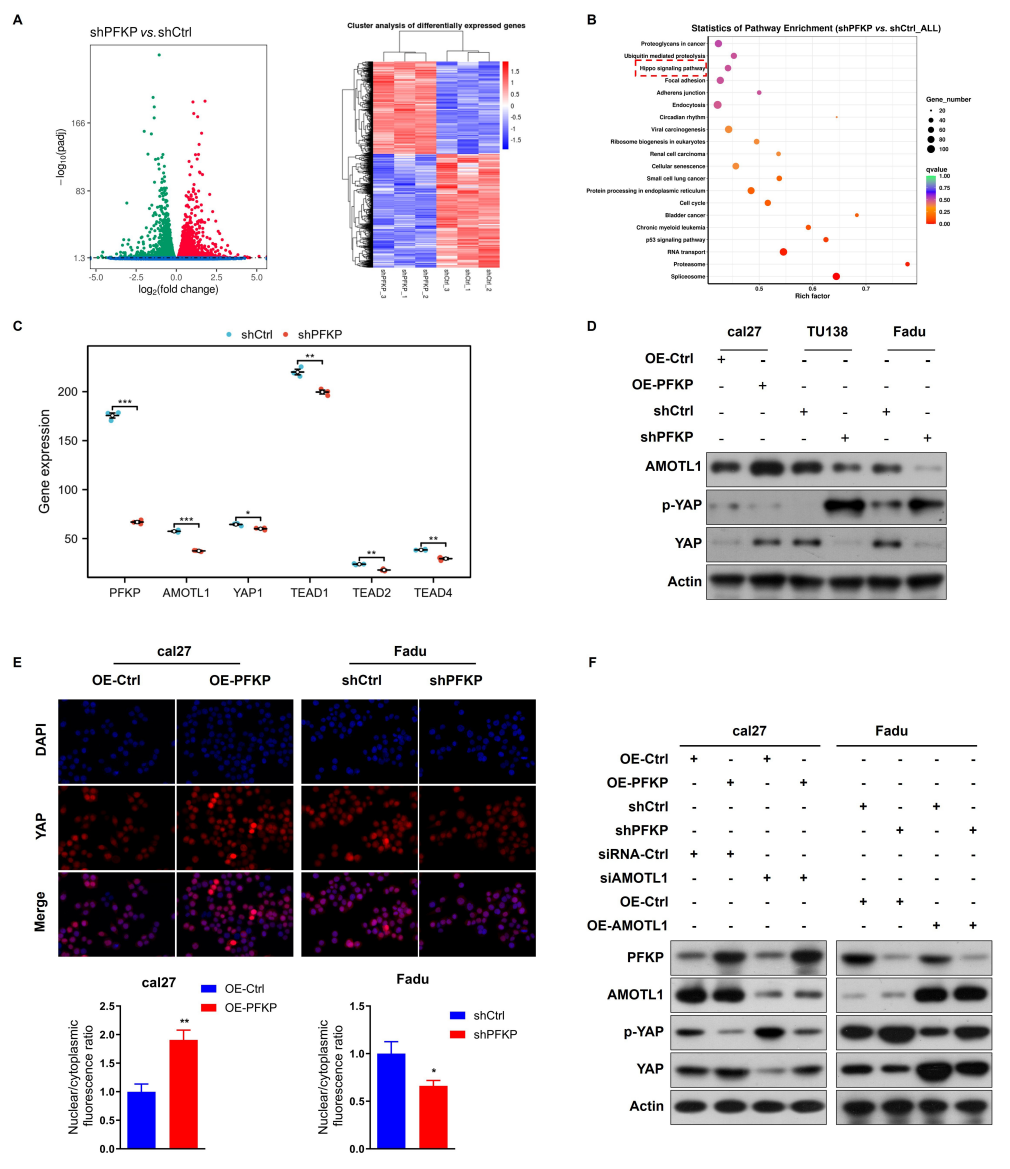
PFKP-mediated nuclear translocation of YAP depends on AMOTL1, leading to Hippo pathway inhibition in HNSCC cells. A. Volcano map and heat map illustrating the differential analysis between knockdown PFKP cells and the control group cells. B. Pathway enrichment analysis of differentially expressed genes. C. Differential expression of AMOTL1, YAP1, and TEADs mRNA after PFKP knockdown. D. Western blot analysis assessing the expression of AMOTL1, YAP, and pYAP upon changes in PFKP expression. E. Immunofluorescence confocal microscopy and quantification of YAP nuclear/cytoplasmic localization used to determine the subcellular localization changes of YAP. F. Evaluation of YAP and p-YAP protein expression under simultaneous or individual changes in PFKP and AMOTL1. Data are presented as mean ± SD. ^*^P < 0.05, ^**^P < 0.01, ^***^P < 0.001.

Transcriptome sequencing was performed after collecting stable knockdown PFKP cells and control group cells. Differential analysis revealed significant enrichment of differentially expressed genes in the Hippo signaling pathway (Figure 5A, B). Knockdown of PFKP resulted in significant downregulation of AMOTL1, YAP1, TEAD 1, TEAD2, and TEAD4 (Figure 5C). AMOTL1 promotes dephosphorylated TAZ/YAP nuclear translocation and forms a transcriptional complex with TEADs to activate downstream target genes, while phosphorylated YAP/TAZ remains in the cytoplasm and undergoes degradation.^[15]^ We hypothesize that PFKP regulates the nuclear transcription of YAP by binding to AMOTL1, thereby affecting HNSCC progression. Western Blot results demonstrated that PFKP overexpression increased AMOTL1 and YAP expression while decreasing p-YAP expression, whereas PFKP knockdown had the opposite effect (Figure 5D). Immunofluorescence confocal microscopy revealed that PFKP overexpression increased nuclear YAP, while PFKP knockdown decreased nuclear YAP (Figure 5E). Simultaneous overexpression of PFKP and knockdown of AMOTL1 reduced YAP expression and increased p-YAP expression compared to PFKP overexpression alone (Figure 5F). Similarly, concurrent knockdown of PFKP and overexpression of AMOTL1 decreased p-YAP expression and increased YAP expression, both of which were rescued (Figure 5F). These findings suggest that PFKP-mediated nuclear translocation of YAP depends on AMOTL1, leading to the inhibition of the Hippo pathway in HNSCC cells.

### PFKP/AMOTL1 enhances aerobic glycolysis and tumor progression in HNSCC relying on nuclear translocation of YAP

**Figure 6:**
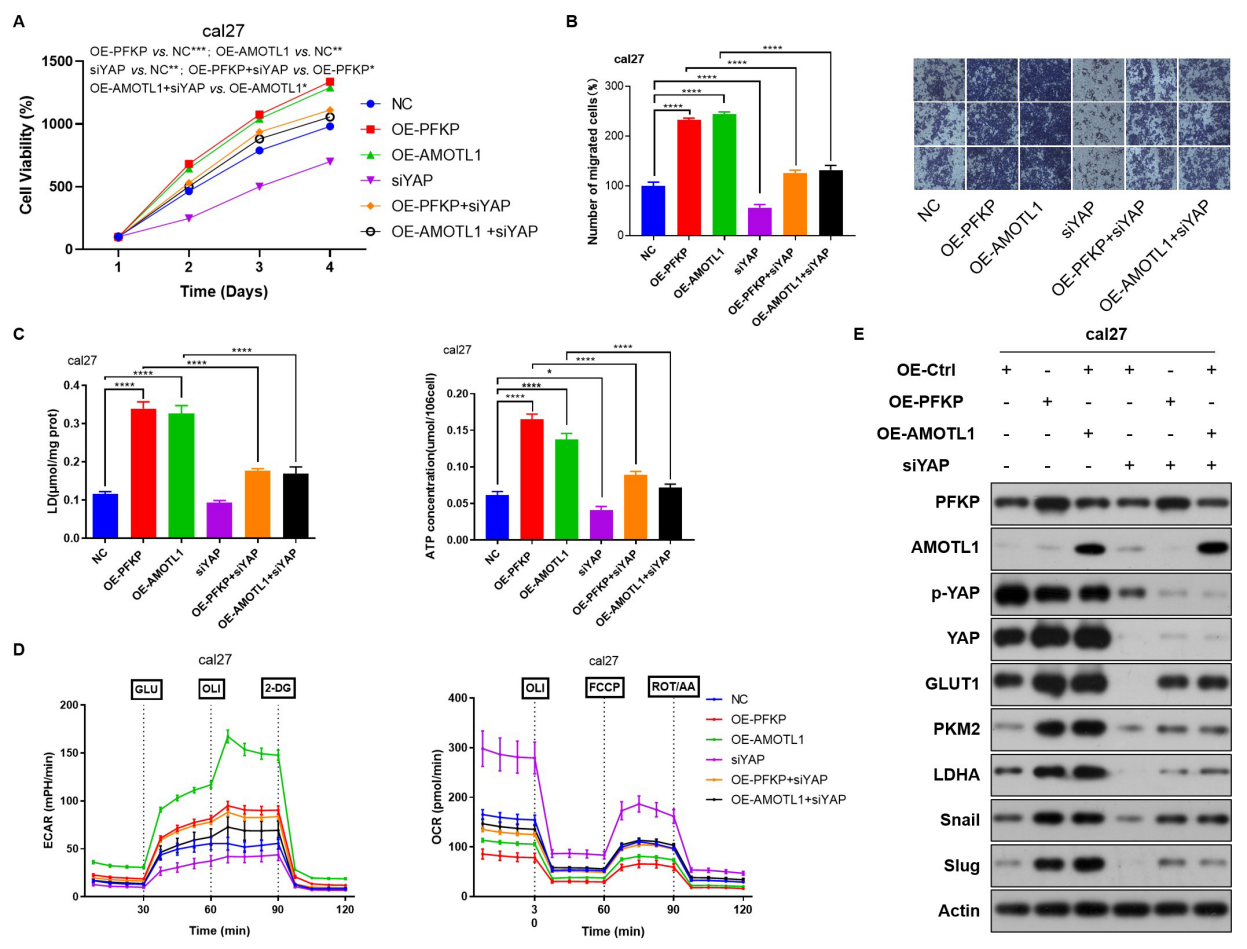
PFKP/AMOTL1 enhances aerobic glycolysis and tumor progression mediated by nuclear translocation of YAP in HNSCC. A. Cell proliferation in cal27 cells was assessed using the CCK-8 assay to investigate the tumor-promoting effect of PFKP and AMOTL1 in the presence of YAP. B. Transwell assays were performed to evaluate the impact of YAP on PFKP and AMOTL1-induced tumor migration. C. Lactate and ATP levels were measured to examine the influence of YAP on PFKP and AMOTL1-induced tumor aerobic glycolysis. D. Western blot analysis was conducted to assess changes in the expression of glycolysis-related proteins (GLUT1, PKM2, LDHA) and EMT-related molecules (Snail and Slug) in response to variations in YAP expression. E. Seahorse metabolic analysis was employed to investigate the role of YAP in mediating aerobic glycolysis induced by PFKP and AMOTL1. Data are presented as mean ± SD. ^*^P < 0.05, ^**^P < 0.01, ^***^P < 0.001, ^****^P < 0.0001.

To investigate the role of PFKP/AMOTL1 binding in tumor progression through transcriptional regulation of YAP, this study conducted overexpression of PFKP combined with simultaneous knockdown of YAP, as well as overexpression of AMOTL1 along with simultaneous knockdown of YAP. CCK8 results demonstrated that knockdown of YAP partially reversed the tumor-promoting function of PFKP/AMOTL1 (Figure 6A). Additionally, the cell migration test revealed that knockdown of YAP could reverse the ability of PFKP/AMOTL1 to promote tumor cell migration (Figure 6B). Compared to the individual overexpression of PFKP or AMOTL1, the co-overexpression of PFKP or AMOTL1 with concurrent knockdown of YAP significantly decreased lactate production, ATP levels, the expression of glycolytic metabolism-related proteins as well as EMT-related molecules (Figure 6C, D). Moreover, Seahorse metabolic analysis results indicated that knockdown of YAP, in conjunction with the overexpression of PFKP or AMOTL1, resulted in the recovery of increased ECAR and decreased OCR (Figure 6E). These findings suggest that knocking down YAP partially reversed the functions of PFKP/AMOTL1 in promoting glycolysis and inhibiting mitochondria in HNSCC. Taken together, these results demonstrate that the downregulation of YAP partially counteracted the proliferative, migratory, EMT and aerobic glycolysis effects of PFKP/AMOTL1.

### Enhanced PFKP drives HNSCC advancement in vivo through AMOTL1/YAP

**Figure 7:**
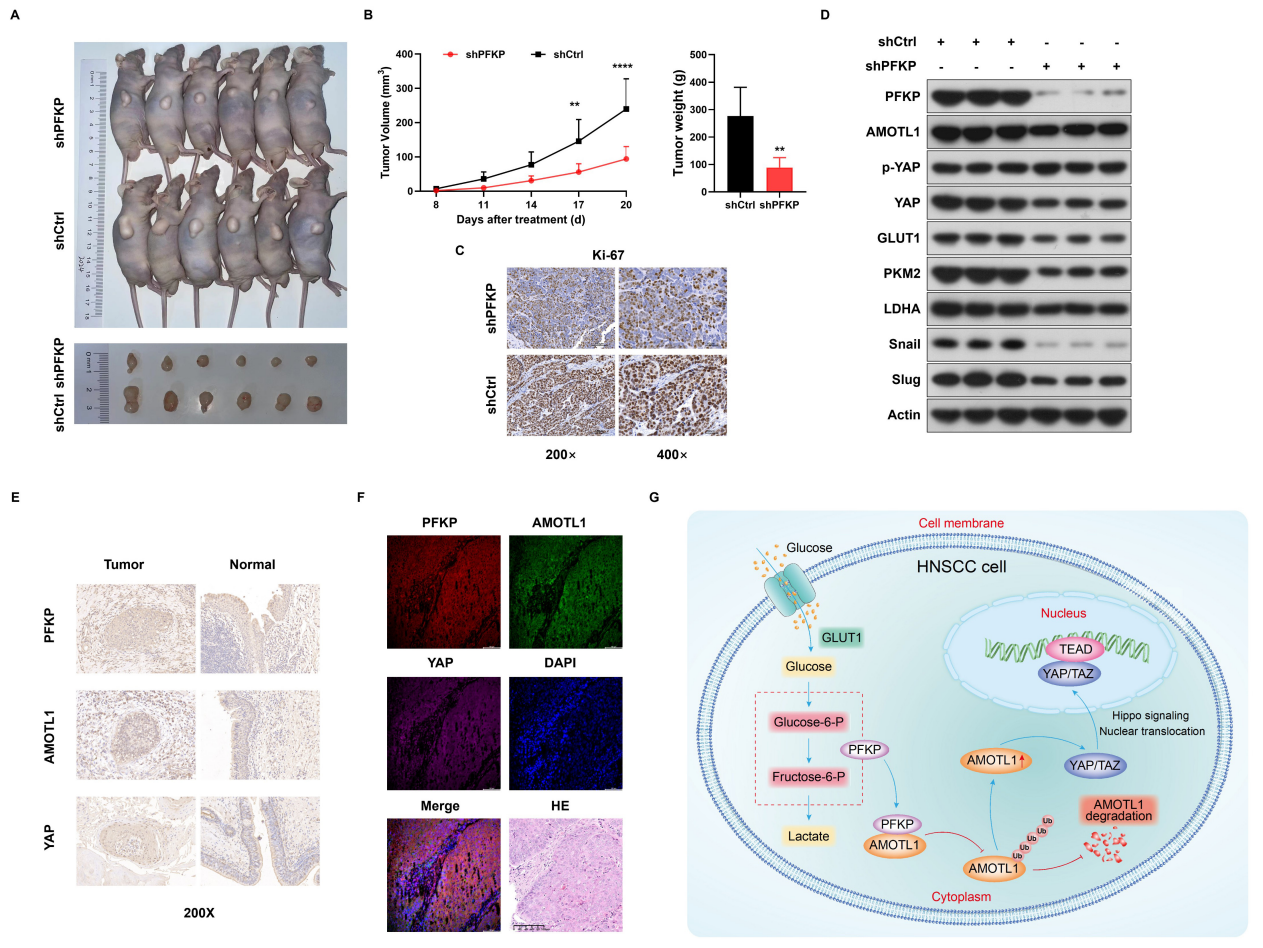
Enhanced PFKP promotes HNSCC progression in vivo via AMOTL1/YAP pathway. A. Tumor growth was monitored after subcutaneous inoculation of designated cells into nude mice. B. Tumor volumes and weights were compared between the PFKP knockdown group and the control group in xenograft tissues of nude mice. C. Representative IHC images of Ki-67 in xenograft tissues are shown. D. Western blot analysis showed changes in the expression of glycolysis-related proteins, EMT-related molecules, AMOTL1, pYAP and YAP after PFKP knockdown. E. IHC analysis of protein PFKP expression differences between cancer and normal tissues. F. The mIHC experiment was used to observe the co-localization of PFKP, AMOTL1 and YAP. G. A schematic diagram depicting a potential mechanism by which PFKP promotes glycolytic metabolism and tumor progression in HNSCC through AMOTL1/YAP/Hippo. Data are presented as mean ± SD. ^**^P < 0.01, ^****^P < 0.0001.

We established nude mouse models by subcutaneously injecting stable PFKP-knockdown TU138 cells and control group single-cell suspensions to investigate the promoting role of PFKP in tumor occurrence and development *in vivo*. Subcutaneous tumors were isolated after sacrificing the animals (Figure 7A). Knockdown of PFKP significantly reduced the volume and weight of subcutaneous tumors compared with the control group (Figure 7B). Additionally, PFKP knockdown led to decreased expression of Ki-67, indicating effective inhibition of HNSCC growth (Figure 7C). Western Blot results showed decreased expression levels of AMOTL1, YAP, glycolysis-related proteins and EMT-related proteins in the PFKP-knockdown group, while p-YAP expression was increased (Figure 7D). These findings support the promotion of glycolysis metabolism and tumor progression by PFKP *via* the AMOTL1/YAP/Hippo *in vivo*. Remarkably, the tumor tissues exhibited elevated PFKP, AMOTL1 and YAP expression levels (Figure 7E). In the mIHC experiment, co-localization of PFKP, AMOTL1 and YAP was observed (Figure 7F). These results indicate that PFKP promotes glycolytic metabolism and tumor progression *in vivo* through AMOTL1/YAP/Hippo.

## Discussion

Tumor cells rely on aerobic glycolysis for energy to support proliferation and survival. In nutrient-deprived environments, they adapt to maintain biosynthesis, energy production, and redox balance.^[16]^ As a key glycolytic enzyme, PFKP enhances aerobic glycolysis, promoting tumor cell proliferation and progression despite metabolic imbalances. However, its role in HNSCC remains unclear. Our study demonstrates that PFKP directly interacts with AMOTL1, inhibiting its ubiquitination and degradation, facilitating YAP nuclear translocation, and inhibiting the Hippo pathway (Figure 7G). These findings position PFKP as a crucial driver of HNSCC progression and a potential therapeutic target.

Our findings that PFKP drives glycolysis and inhibits the Hippo pathway through AMOTL1/YAP have significant implications that extend beyond direct tumor cell proliferation and migration. The resulting metabolic reprogramming and epithelial plasticity likely contribute critically to the immunosuppressive and therapy-resistant nature of HNSCC. Firstly, the PFKP-driven glycolytic flux and lactate production would acidify the TME, a condition known to impair anti-tumor immunity by inhibiting T-cell function and recruitment, potentially affecting the efficacy of immunotherapies such as anti-PD-L1/anti-TGF-β bispecific antibodies.^[13]^ This provides a rationale for exploring PFKP inhibition as a strategy to reverse immunosuppression and synergize with existing immunotherapies, a synergy concept supported by recent advances in combinational regimens.^[17]^ Secondly, the activation of YAP and the resultant EMT program are key drivers of radioresistance and metastasis. The convergence of PFKP signaling on pathways also activated by other HNSCC risk factors, such as the MEK/ERK pathway implicated in nicotine-induced progression,^[14]^ underscores the central role of PFKP in a common pro-tumorigenic network. Finally, the regulation of epithelial plasticity by PFKP/AMOTL1/YAP mirrors mechanisms of mucosal remodeling observed in other epithelial pathologies, such as the MEK and WNT-driven processes in chronic rhinosinusitis,^[18]^ reinforcing the broad relevance of pathways controlling epithelial cell state in disease progression.

Transcription factor YY1 directly binds to and activates PFKP, promoting tumor aerobic glycolysis and malignant progression.^[19,20]^ In glioblastoma, PFKP plays a key role in activating VEGF expression, stimulating angiogenesis, and driving tumor growth.^[21]^ Notably, Wang *et al*. identified PFKP as a nucleocytoplasmic shuttling protein, dependent on c-Myc to upregulate CXCR4 expression in T-cell lymphoma, with nuclear PFKP accumulation linked to poor patient survival.^[22]^ In contrast, Liu *et al*. found PFKP mainly localized in the cytoplasm, where a positive feedback loop with c-Myc accelerates HNSCC progression.^[23]^ However, PFKP’s role in HNSCC aerobic glycolysis remains unexplored. Our study demonstrates significant PFKP overexpression promotes tumor progression by driving EMT and aerobic glycolysis in HNSCC.

Using Co-IP, we identified a direct interaction between PFKP and AMOTL1. The AMOT family, which includes AMOT (p80 and p130 subtypes), AMOTL1, and AMOTL2, consists of angiostatin-binding proteins highly expressed in tumor cells. Previous studies have shown that HOXD 10 promotes proliferation, adhesion, and migration in HNSCC by regulating AMOT-p80 expression.^[24]^ However, the role of AMOTL1 in HNSCC has not been explored. Our findings reveal that PFKP promotes EMT and aerobic glycolysis in HNSCC cells in an AMOTL1-dependent manner. Ubiquitination is the most common post-translational modification of AMOT proteins. AMOTL2, for example, is ubiquitinated at K347 and K408, targeted by USP9X, and interacts with LATS kinase in the Hippo pathway, making it a potential therapeutic target.^[25]^ In our study, we found that PFKP inhibits AMOTL1 ubiquitination, preventing its degradation *via* the proteasome. The NEDD4 family of HECT-domain E3 ligases, is a known regulator of the related protein AMOT and other components of the Hippo pathway.^[26,27]^ We hypothesize that ITCH or a related E3 ligase is a strong candidate for mediating AMOTL1 ubiquitination. We speculate that the direct binding of PFKP to AMOTL1 might sterically hinder the interaction between AMOTL1 and its E3 ligase, thereby protecting AMOTL1 from ubiquitin-mediated proteasomal degradation. the identification and functional validation of the specific E3 ubiquitin ligase constitute a major direction of our future work.

AMOTs play a crucial role in the Hippo signaling pathway by regulating the nuclear translocation of YAP.^[15,28]^ Interestingly, AMOTs have a dual role in YAP regulation: they can either bind directly to YAP or modulate its phosphorylation, influencing downstream gene expression. As an auxiliary factor, AMOT can enhance YAP activity by inhibiting its phosphorylation, promoting tumorigenesis.^[29]^ However, other studies suggest that AMOT acts as a scaffold protein, sequestering YAP in the cytoplasm or at cell junctions and facilitating LATS1/2-mediated YAP phosphorylation.^[30]^ Phosphorylation of AMOT at S176 directs YAP to the plasma membrane, while AMOT-p130 promotes YAP nuclear localization and enhances its transcriptional activity.^[31,32]^ We found that the PFKP-bound form of AMOTL1 functions as a YAP activator in our HNSCC model. We hypothesize that binding to PFKP is the pivotal switch that converts AMOTL1 into a YAP activator. We speculate that PFKP, a cytoplasmic protein, may sequester AMOTL1 away from membrane complexes where it could act as a inhibitor. Alternatively, the PFKP-AMOTL1 interaction may induce a conformational change that masks AMOTL1’s inhibitory domain or promotes its association with nuclear transport machinery. In the future, we need more detailed experimental designs to verify our hypothesis. And it is important to note that the therapeutic potential of targeting PFKP, particularly in combination with YAP pathway inhibitors, remains to be empirically validated in future preclinical studies using specific pharmacological agents.

## Conclusions

In conclusion, this study elucidates a novel mechanism whereby PFKP directly interacts with AMOTL1, promoting the nuclear translocation of YAP, thereby inhibiting Hippo pathway to drive HNSCC EMT and aerobic glycolysis. Our work delineates a novel PFKP/AMOTL1/YAP signaling axis that drives HNSCC progression, thereby providing a strong mechanistic rationale for future investigations into targeting this pathway.

## Supplementary Material

Supplementary Material Details
