# Supplementary material for: PFKP binding AMOTL1 promotes tumor aerobic glycolysis and epithelial-mesenchymal transition by modulating Hippo pathway in head and neck cancer
Source: J Transl Int Med. 2026 Feb 13;14(1):108–22. doi: 10.1515/jtim-2026-0006 (PMC12916276; doi:10.1515/jtim-2026-0006)
Supplement: Supplementary file 1 — Supplementary Material Details [file jtim-2026-0006_sm.pdf]

Supplementary Materials

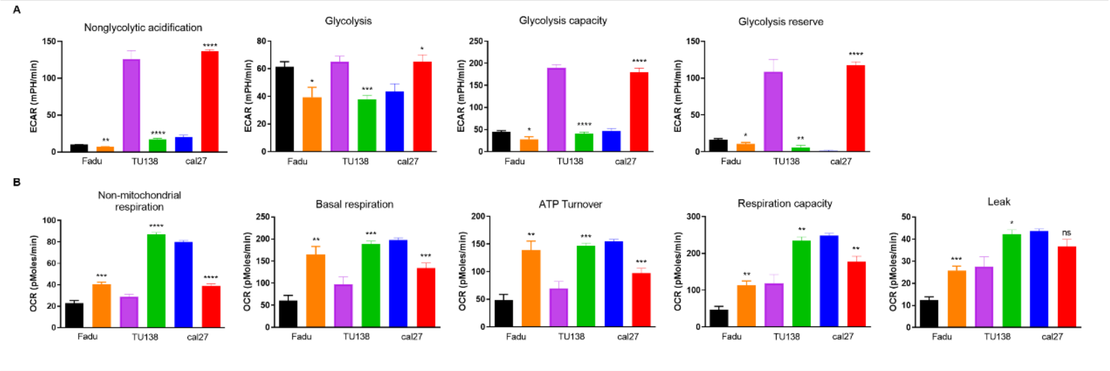

Supplementary Figure S1: Seahorse metabolic analysis after PFKP expression changes.

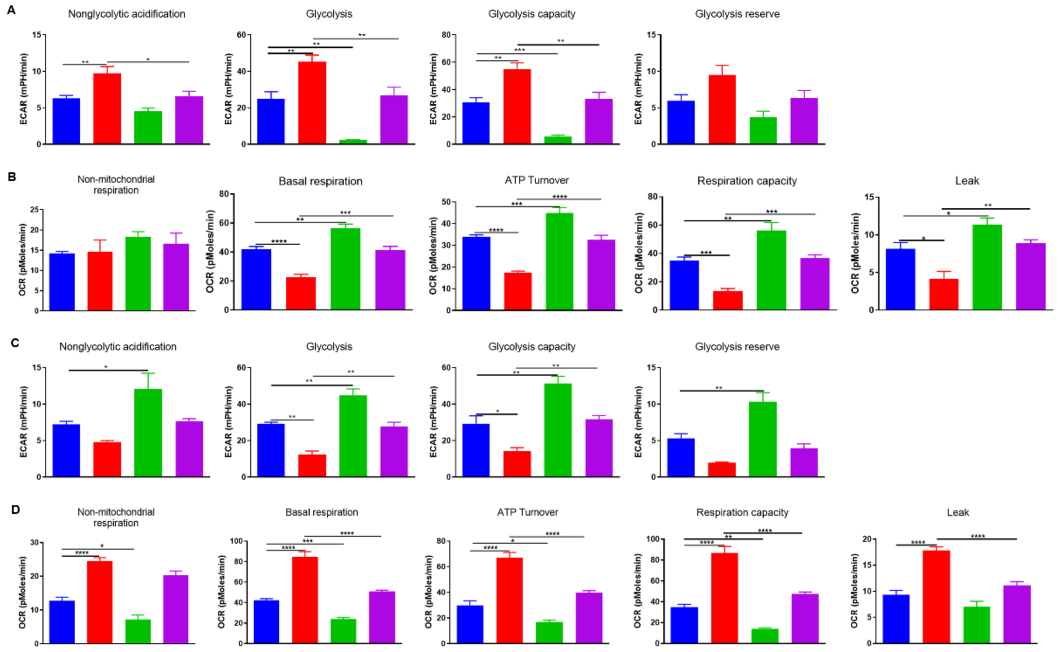

Supplementary Figure S2: Seahorse metabolic analysis after simultaneous overexpression of PFKP and knockdown of AMOTL1, or knockdown of PFKP alongside overexpression of AMOTL1.

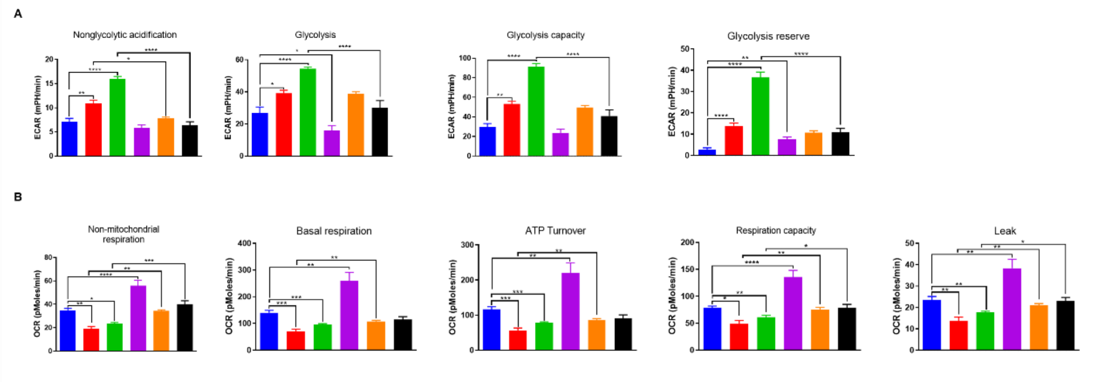

Supplementary Figure S3: Seahorse metabolic analysis after co-overexpression of PFKP and AMOTL1

with simultaneous YAP knockdown.

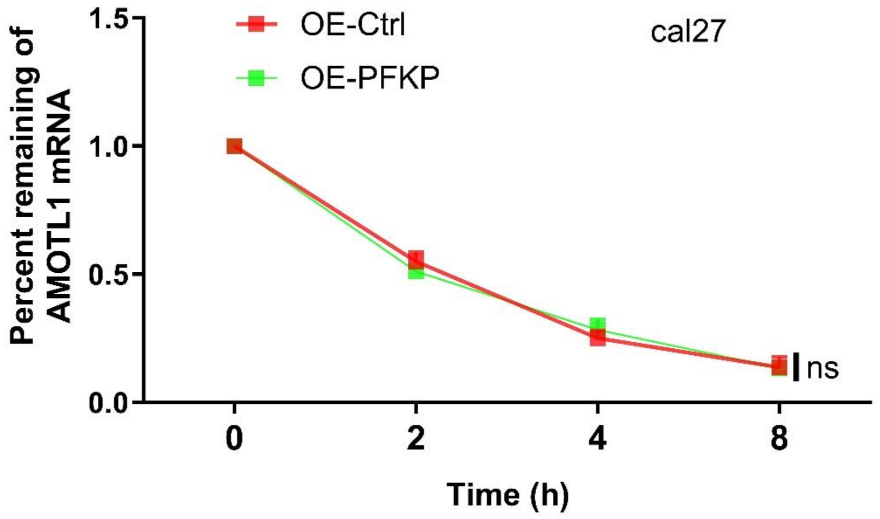

Supplementary Figure S4: PFKP does not affect AMOTL1 mRNA stability in HNSCC cells.

Supplementary Table S1: List of antibodies.

Antibodies	Source	Catalog number	Dilution
rabbit anti-PFKP	Proteintech	13389-1-AP	1:1000
rabbit anti-GLUT1	Abcam	ab115730	1:2000
rabbit anti-PKM2	Abcam	ab137852	1:1000
rabbit anti-LDHA	Cell Signaling Technology	CST3582	1:1000
rabbit anti-Snail	Cell Signaling Technology	CST3879T	1:500
mouse anti-Slug	Abcam	ab51772	1:500
rabbit anti-AMOTL1	Proteintech	16871-1-AP	1:1000
rabbit anti-p-YAP	ABclone	AP0489	1:1000
rabbit anti-YAP	Proteintech	13584-1-AP	1:2000

mouse anti-IgG	Cell Signaling Technology	7076	1:5000
rabbit anti-Ub	Proteintech	10201-2-AP	1:5000
Ki-67	Abcam	Ab16667	1:200

Supplementary Table S2: Clinicopathological characteristics of the 51 HNSCC patients.

Characteristics	*N* (%)
**Sex**	
M	50
F	1
**Smoking history**	
Yes	43
No	8
**T Stage**	
T2	19
T3	22
T4	10
**N Stage**	
N0	30
N1	6
N2	15
**Clinical Stage**	

II	11
III	20
IV	20

Supplementary WB Original Images

Figure 2F

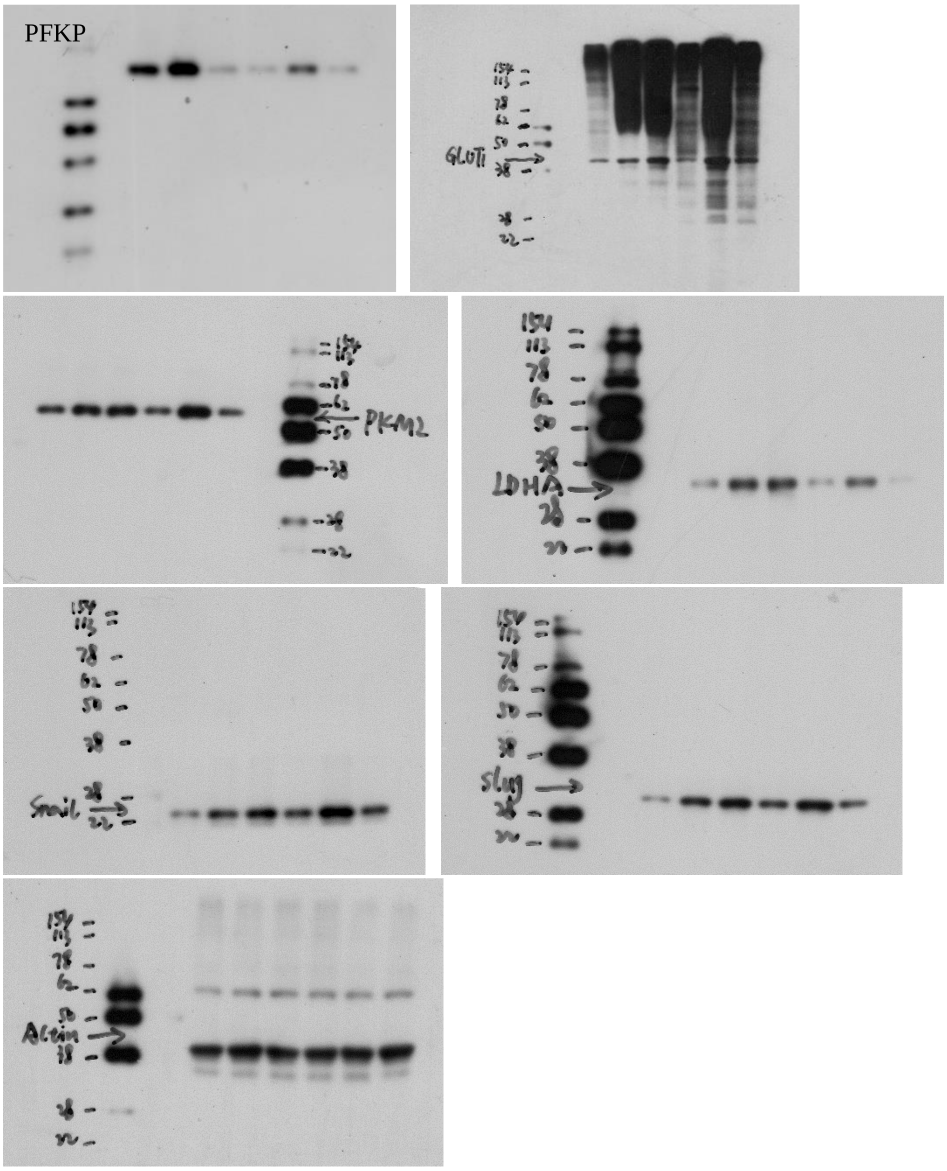

Figure 3B

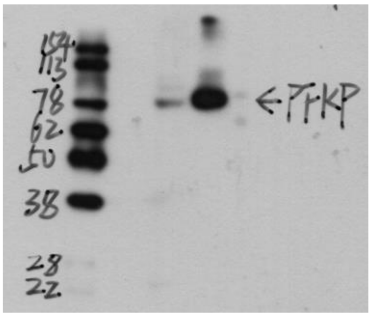

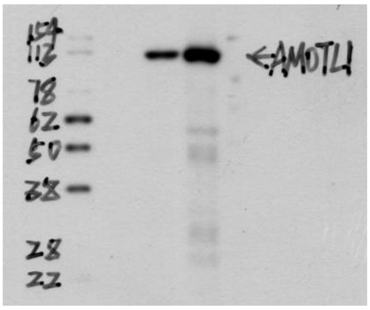

cal27 Input

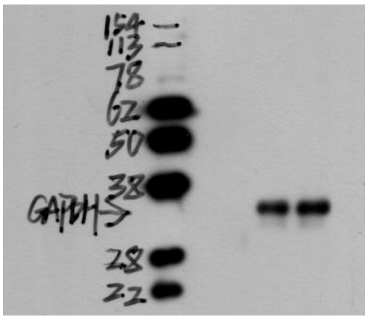

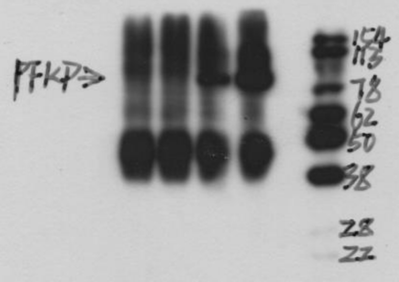

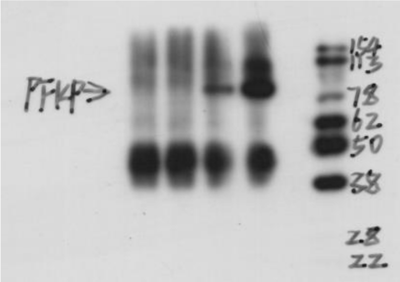

IP

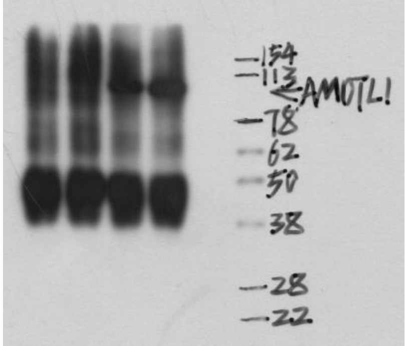

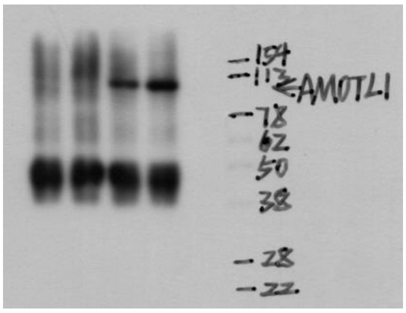

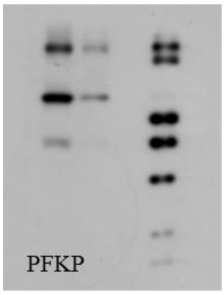

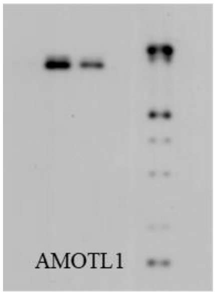

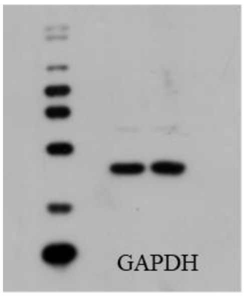

Fadu

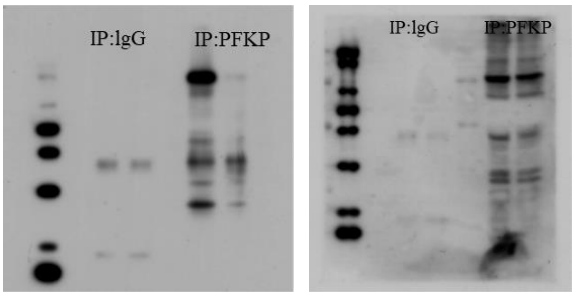

Figure 3C

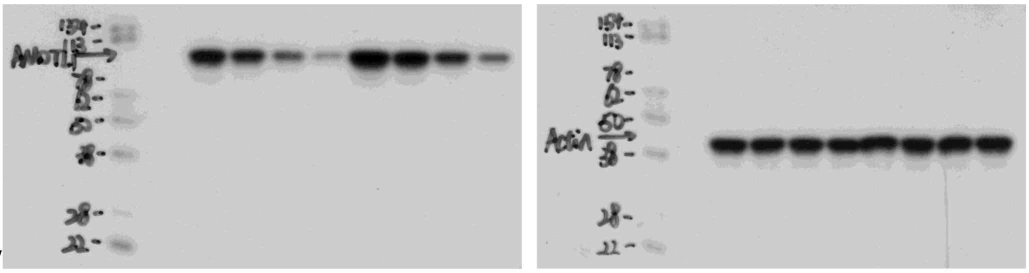

cal27

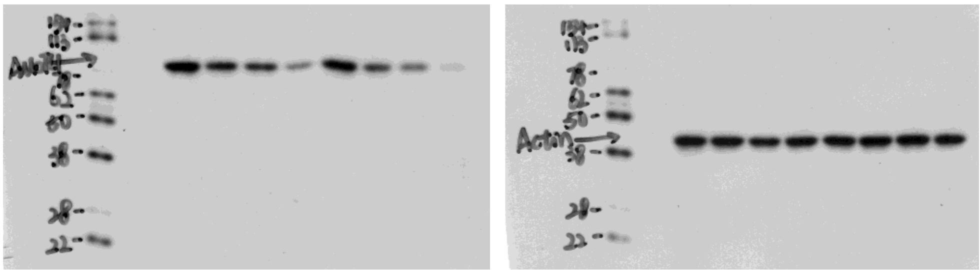

Fadu

Figure 3D

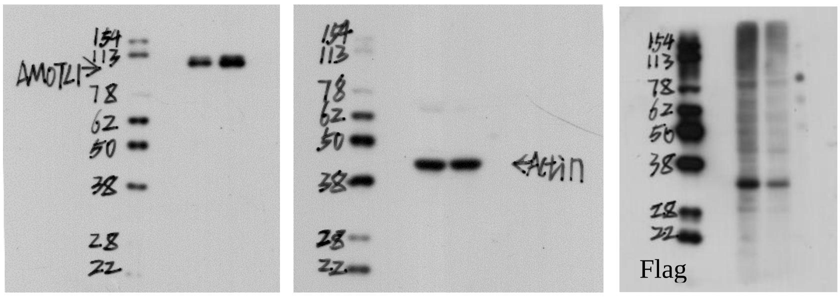

cal27

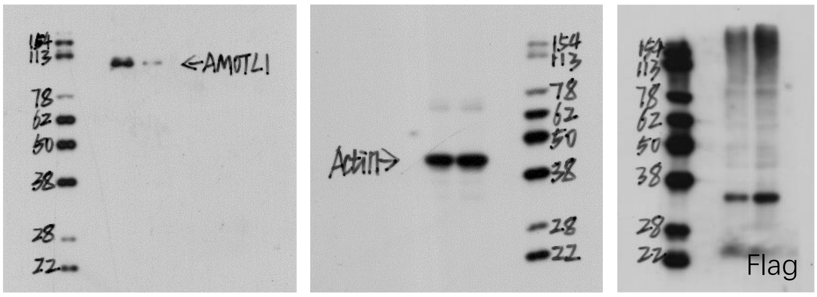

Fadu

Figure 4D

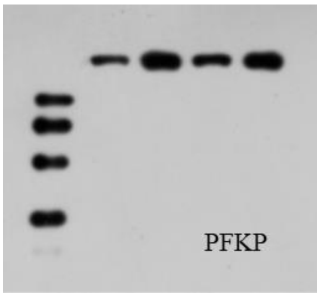
PFKP
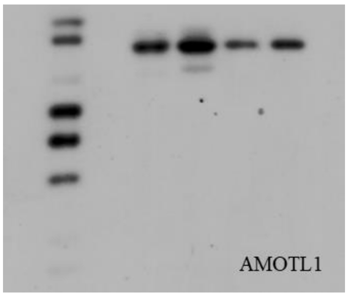
AMOTL1
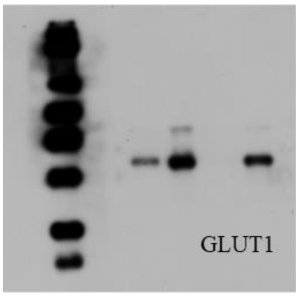
GLUT1

cal27

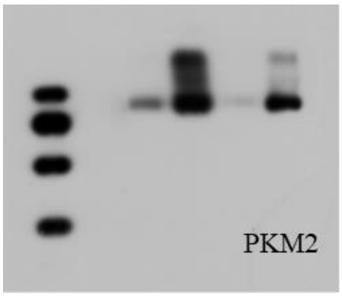
PKM2
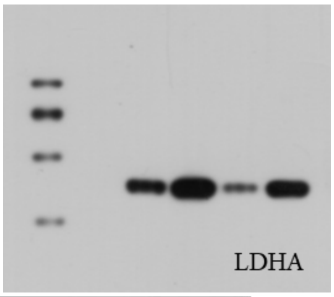
LDHA
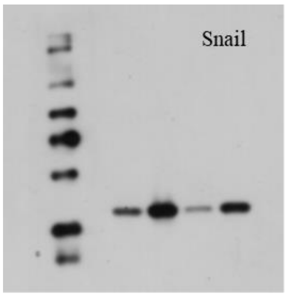
Snail
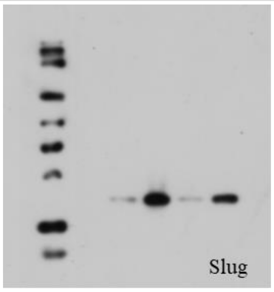
Slug
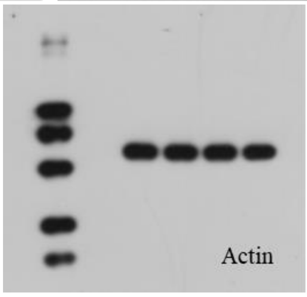
Actin
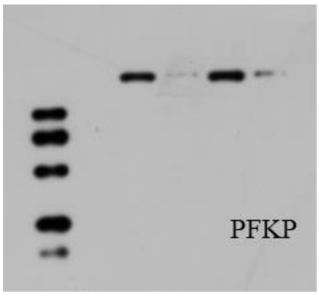
PFKP
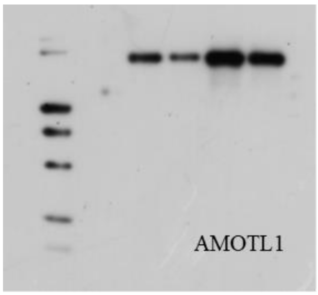
AMOTL1
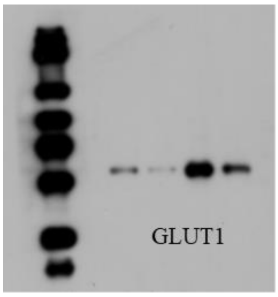
GLUT1

Fadu

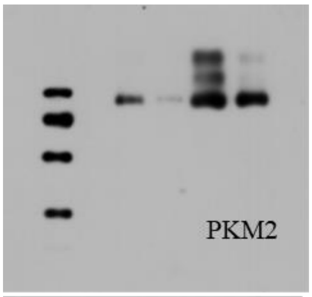
PKM2
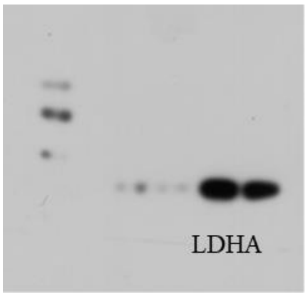
LDHA
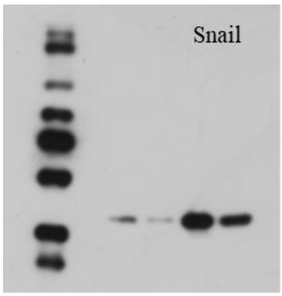
Snail
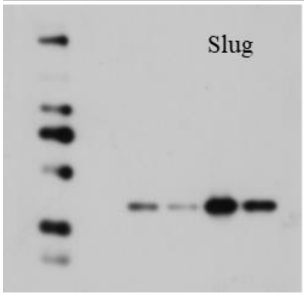
Slug
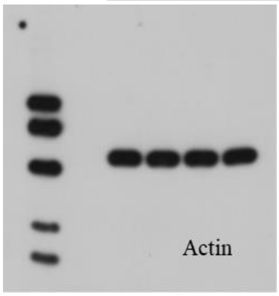
Actin

Figure 5D

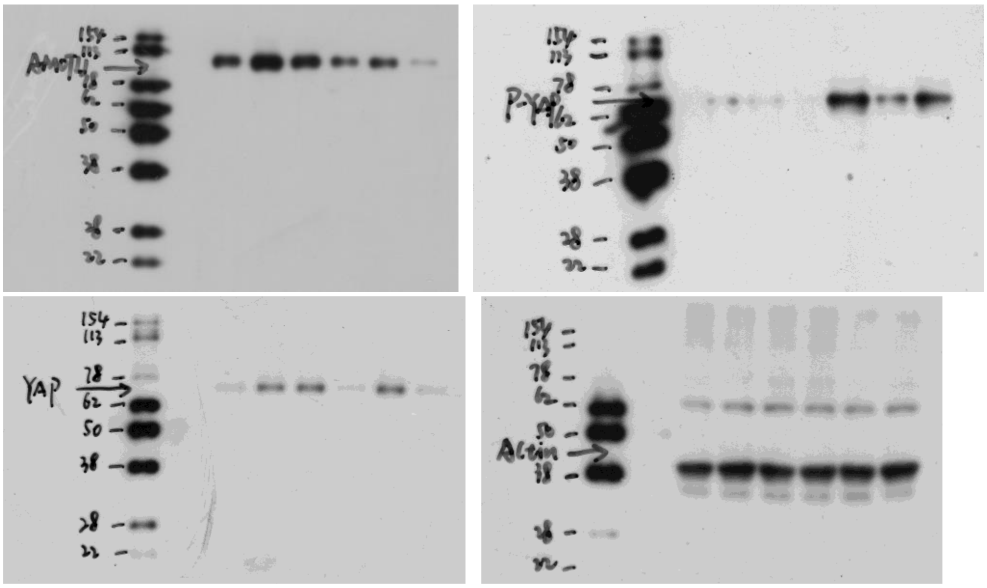

Figure5 F

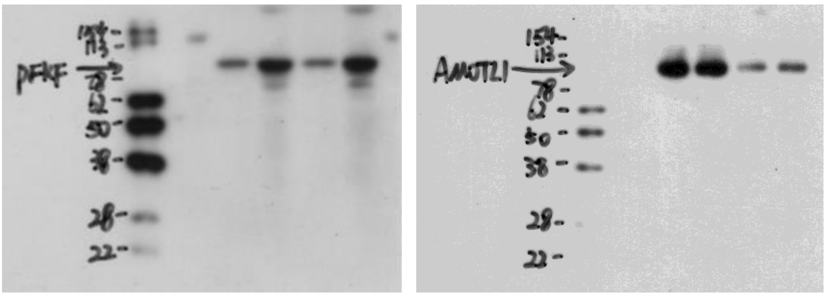

cal27

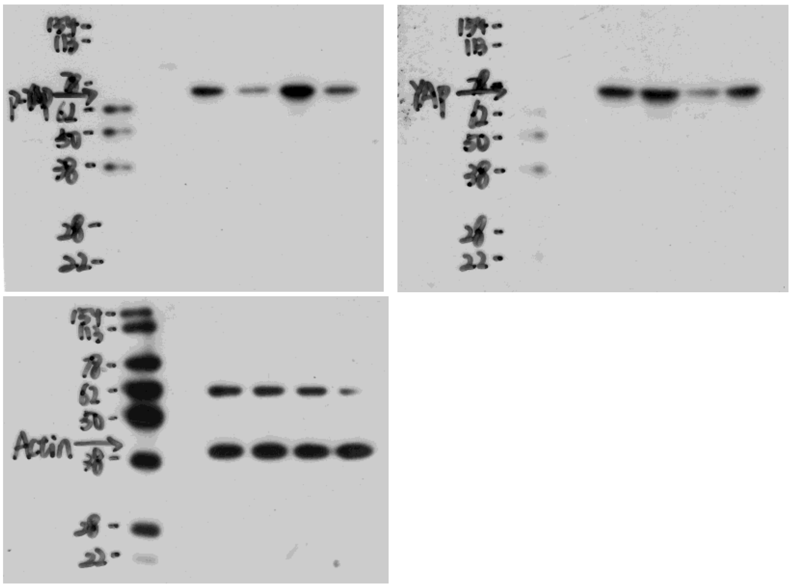

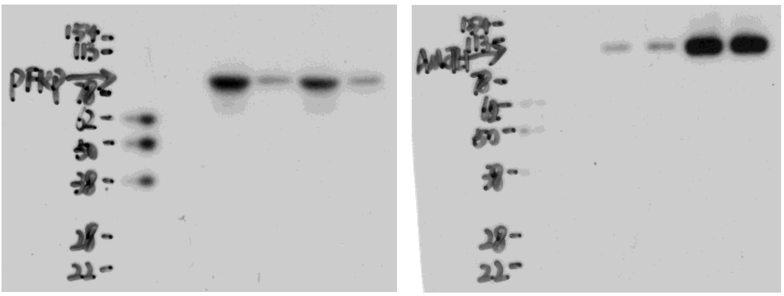

Fadu

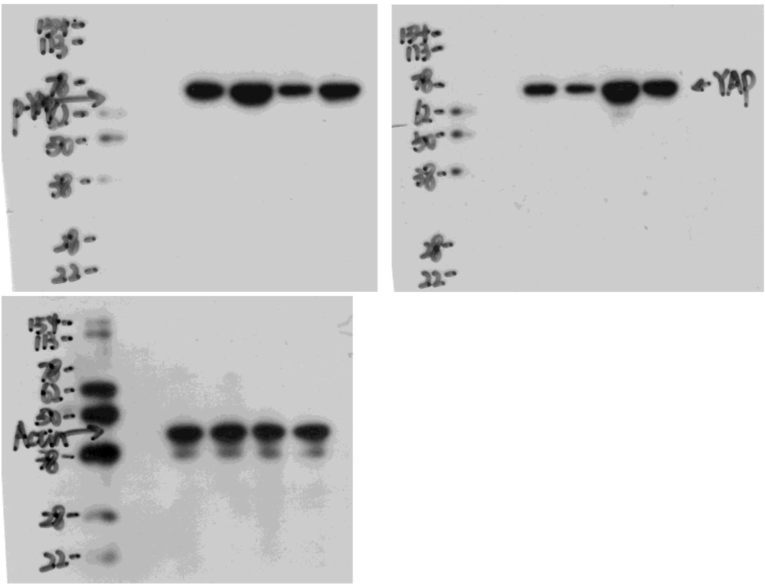

Figure6 E

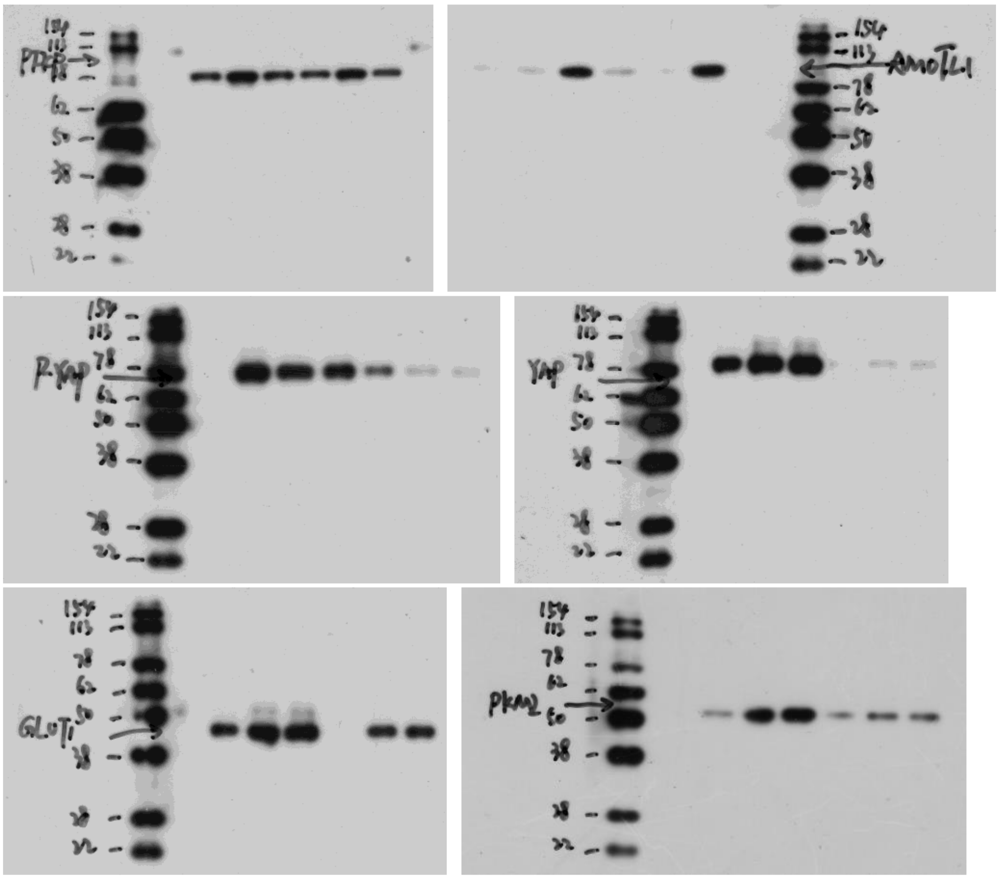

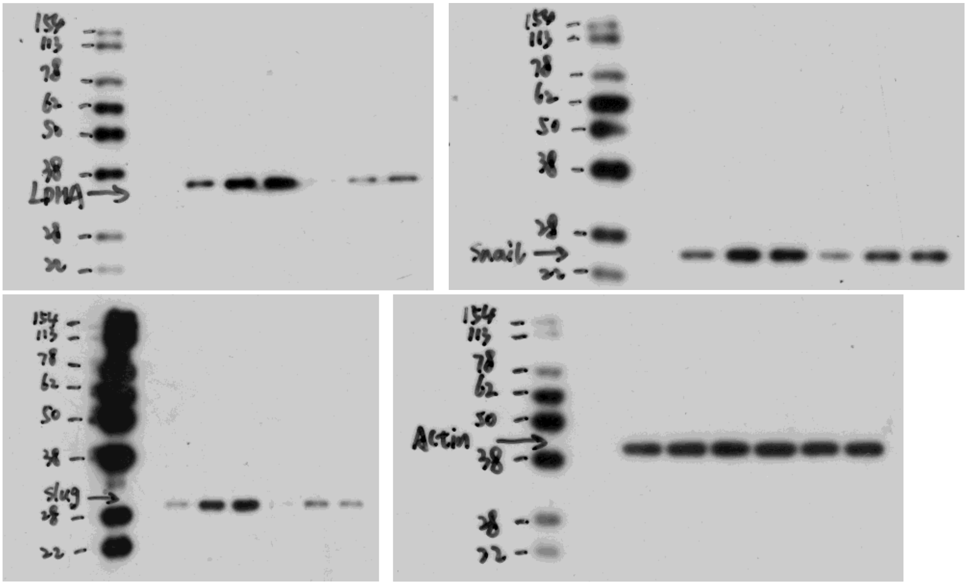
LDHA
Snail
Slug
Actin

Figure7D

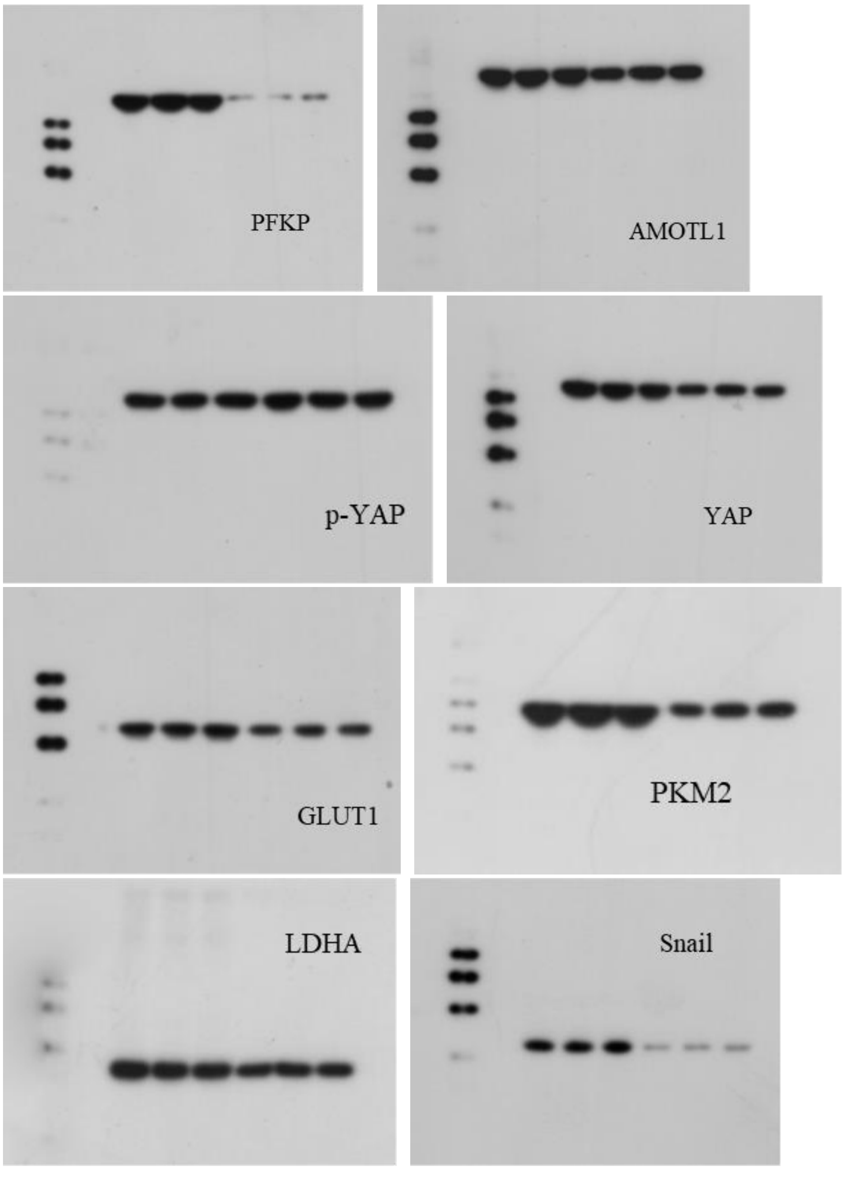
PFKP
AMOTL1
p-YAP
YAP
GLUT1
PKM2
LDHA
Snail

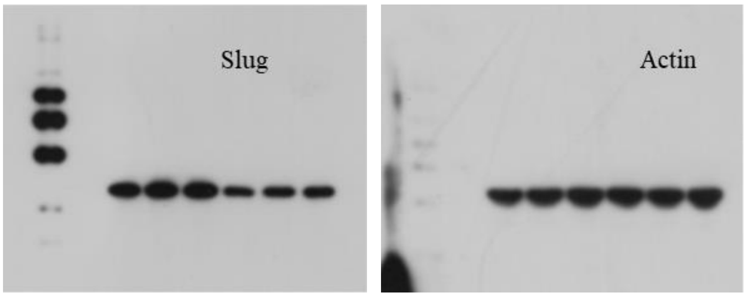
Slug
Actin
